# Supplementary material for: Clusters of Physical Frailty and Cognitive Impairment and Their Associated Comorbidities in Older Primary Care Patients
Source: Healthcare (Basel). 2021 Jul 15;9(7):891. doi: 10.3390/healthcare9070891 (PMC8304880; doi:10.3390/healthcare9070891)
Supplement: Supplementary file 1 [file healthcare-09-00891-s001.zip › healthcare-1278702-supplementary.pdf]

Table 1

Descriptive statistics of numerical variables

Parameter	Min	Max	Median (interquartile range)	Mean (standard deviation)
Age (years)	60	90	71 (10)	71.11 (6.36)
BMI (kg/m^2)	19.02	46.05	29.67 (5.73)	30.19 (4.49)
Waist circumference (cm)	50.00	143.00	99.00 (16)	99.91 (12.01)
Mid arm circumference (cm)	18.00	45.00	31.00 (5)	31.41 (3.59)
A total number of diagnoses	0	10	3 (2)	3 (1.79)
A total number of prescribed medications	0	15	3 (3)	4 (2.15)
A total number of medications with effect on mental functions	0	9	3 (2)	3 (1.67)
A total number of sensory/functional disorders	0	5	2 (2)	2 (1.05)
Fasting glucose (mmol/L)	3.60	16.20	5.60 (1.7)	6.24 (1.94)

Total cholesterol (mmol/L)	2.90	9.70	5.70 (1,7)	5.76 (1.34)
LDL cholesterol (mmol/L)	1.20	8.90	3.50 (1.4)	3.58 (1.20)
HDL cholesterol (mmol/L)	0.60	2.30	1.40 (0.4)	1.39 (0.32)
Triglycerides (mmol/L)	0.60	7.70	1.70 (09)	1.82 (0.94)
Glomerular filtration rate (mL/min/1.73 m^2)	18.00	191.00	85.00 (37)	86.42 (26.84)
C-reactive protein (mg/L)	0.20	25.00	2.20 (3.2)	3.56 (3.98)
Haemoglobin (g/L)	54.00	177.00	138.00 (15)	137.10 (13.46)
Erythrocyte number (x 10^{12}/L)	2.70	5.87	4.59 (0.5)	4.57 (0.43)

Table 2

Descriptive statistics of categorical variables

Parameter	Values (%)
Gender	M – 35<br>F – 65
Hypertension	Yes (< 10 years) - 33<br>Yes (≥ 10 years) - 45<br>No - 22
Diabetes mellitus type 2	Less (< 5 years) - 10<br>More (≥ 5 years) - 13<br>No - 77
Chronic obstructive pulmonary disease	Yes - 5<br>No - 95
Asthma or allergic rhinitis	Yes - 8<br>No - 92
Chronic heart disease (failure)	Yes- 15<br>No - 246
Coronary artery disease	Yes - 12<br>No - 88
Cerebrovascular disease	Yes - 7<br>No - 93
Periphery artery disease	Yes - 3<br>No - 97
Upper gastro-intestinal tract disorders	Yes - 43<br>No - 57
Chronic hepatic disorders	Yes – 0.03<br>No – 99.97
Malignant disease (excluding terminal phase and patients under active treatment, also skin tumors)	Yes - 11<br>No - 89
Osteoporosis (diagnosis confirmed)	Yes - 10

	No - 90
Severe osteoarthritis (OA of major ankles - 3 or more attacks a year or deformities or continuous pain) or dg of rheumatic arthritis	Yes - 39<br>No - 61
Syndroma lumbale (long lasting or frequently repeated)	Yes - 39<br>No - 61
Parkinson`s disease	Yes - 2<br>No - 98
Urogenital diseases (chronic cystitis/ pyelonephritis, hyperplasio prostatae – M)	Yes - 13<br>No - 87
Thyroid gland disorders	Yes - 6<br>No - 94
Anxiety/depression (excluding psychosis and dementias)	Yes - 46<br>No - 54
Incontinent and other urinary bladder disorders (having a urinary catheter, taking pads or anamnesis of urinary bladder disorders)	Yes - 5<br>No - 95
Significant visus loss	Yes - 86<br>No - 14
Registered hearing impairment or communication difficulties due to hearing loss	Yes - 26<br>No - 74
Experienced falls	A fall with a fracture - 3<br>A fall without fracture - 25<br>No - 72
Walking with support or visible impaired	Yes - 7<br>No - 93
Complaints on chronic pain	Yes - 39<br>No - 61
Dg of chronic diseases	<3 - 107<br>>=3 - 154
